# Comparative genomics and biological characterization of sequential *Pseudomonas aeruginosa* isolates from persistent airways infection

**DOI:** 10.1186/s12864-015-2276-8

**Published:** 2015-12-29

**Authors:** Irene Bianconi, Julie Jeukens, Luca Freschi, Beatriz Alcalá-Franco, Marcella Facchini, Brian Boyle, Antonio Molinaro, Irena Kukavica-Ibrulj, Burkhard Tümmler, Roger C. Levesque, Alessandra Bragonzi

**Affiliations:** Infections and Cystic Fibrosis Unit, Division of Immunology, Transplantation and Infectious Diseases, San Raffaele Scientific Institute, Milano, Italy; Institut de biologie intégrative et des systèmes (IBIS), Université Laval, Quebec, Canada; Università di Napoli Federico II, Napoli, Italy; Medizinische Hochschule Hannover, Hannover, Germany

**Keywords:** Cystic fibrosis, *P. aeruginosa*, Genome, Adaptation, Chronic infection, Mouse model

## Abstract

**Background:**

*Pseudomonas aeruginosa* establishes life-long chronic airway infections in cystic fibrosis (CF) patients. As the disease progresses, *P. aeruginosa* pathoadaptive variants are distinguished from the initially acquired strain. However, the genetic basis and the biology of host-bacteria interactions leading to a persistent lifestyle of *P. aeruginosa* are not understood. As a model system to study long term and persistent CF infections, the *P. aeruginosa* RP73, isolated 16.9 years after the onset of airways colonization from a CF patient, was investigated. Comparisons with strains RP1, isolated at the onset of the colonization, and clonal RP45, isolated 7 years before RP73 were carried out to better characterize genomic evolution of *P. aeruginosa* in the context of CF pathogenicity.

**Results:**

Virulence assessments in disease animal model, genome sequencing and comparative genomics analysis were performed for clinical RP73, RP45, RP1 and prototype strains. In murine model, RP73 showed lower lethality and a remarkable capability of long-term persistence in chronic airways infection when compared to other strains. Pathological analysis of murine lungs confirmed advanced chronic pulmonary disease, inflammation and mucus secretory cells hyperplasia. Genomic analysis predicted twelve genomic islands in the RP73 genome, some of which distinguished RP73 from other prototype strains and corresponded to regions of genome plasticity. Further, comparative genomic analyses with sequential RP isolates showed signatures of pathoadaptive mutations in virulence factors potentially linked to the development of chronic infections in CF.

**Conclusions:**

The genome plasticity of *P. aeruginosa* particularly in the RP73 strain strongly indicated that these alterations may form the genetic basis defining host-bacteria interactions leading to a persistent lifestyle in human lungs.

**Electronic supplementary material:**

The online version of this article (doi:10.1186/s12864-015-2276-8) contains supplementary material, which is available to authorized users.

## Background

The opportunistic pathogen *Pseudomonas aeruginosa* has broad capabilities to thrive in diverse ecological niches and to establish serious human infections [1]. Poor clinical outcome of *P. aeruginosa*-associated infection was described in immunocompromised patients and those in intensive care units, connected to mechanical ventilation or other invasive devices. *P. aeruginosa* is also the leading cause of chronic lung infections and death in patients with cystic fibrosis (CF), as well as a frequent cause of exacerbations in individuals with advanced chronic obstructive pulmonary disease (COPD) [2].

The genetic basis of *P. aeruginosa* leading to acute or chronic infection is not yet understood [3]. Genome sequencing projects are underway with the aim of providing new data to dissect the molecular basis of *P. aeruginosa* infections. Seventeen completely sequenced and assembled genomes are currently available and draft genomes exist for 561 additional genomes. The genome size of *P. aeruginosa* is larger than those of most sequenced bacteria and varies between 5.2 and 7 Mbp, with ~5500 ORFs [4]. A significant number (8,4 %) of *P. aeruginosa* genes are predicted to be involved in regulation, which at the time of publication was the largest fraction of regulators among sequenced bacterial genomes. Irrespective of their origin, *P. aeruginosa* isolates share a remarkable amount of similarity in their genome content and in virulence traits (core genome). The extent of divergence between strains is determined by extra-chromosomal elements like plasmids or blocks of DNA inserted into the chromosome at various loci [5]. These genetic features are likely to be acquired by horizontal gene transfer from different sources including other species or genera and can be present in subgroups of the *P. aeruginosa* population but may also be unique to single strains, accounting for most of intra- and inter-clonal *P. aeruginosa* genome diversity. These strain-specific segments of the genome are not scattered randomly through the core genome; rather, they tend to be clustered in certain loci, referred to as regions of genome plasticity (RGPs) [6]. The genetic sequences occupying many RGPs are often referred to as genomic islands (GIs) and islets. Therefore, the *P. aeruginosa* chromosome presents a picture of a mosaic, consisting of a conserved core component, interrupted in each strain by the inserted parts of the accessory genome. Genetic elements within the accessory genome may encode properties that contribute to niche-specific adaptation of the particular strains that harbor them.

Furthermore, mutations of single nucleotides also confer specific *P. aeruginosa* phenotypes that are advantageous under certain conditions [7–10]. Long-term colonization of the CF host is maintained by *P. aeruginosa* pathoadaptive lineages, which are clonal with the initially acquired strain and carry phenotypic variants. Pathoadaptive mutations are frequent in virulence genes, essential for acute infection but no longer compatible with the novel lifestyle of the *P. aeruginosa* in CF airways. However, little is known about the genetic basis and the biology of host-bacteria interactions leading to a persistent lifestyle of *P. aeruginosa*.

To define the genetic basis of *P. aeruginosa* persistent lifestyle, longitudinal isolates from CF patient were selected. In particular, *P. aeruginosa* RP73 was isolated after long-term chronic infection and compared with the preceding RP1 and clonal RP45, as well as prototype PAO1 and PA14 strains. When murine model of chronic lung infection was used, RP73 showed a marked persistent lifestyle. Thus, genome sequencing and comparative genomics analysis were carried out. Our results show the links between genomic properties and pathogenic potential of RP73 that may define the basis of long-term chronic infection by *P. aeruginosa*. The significance of these results is discussed in the context of understanding disease pathogenesis.

## Results and discussion

### Chronic colonization of a CF patient’s airways with the P. aeruginosa RP isolates

**Fig. 1:**
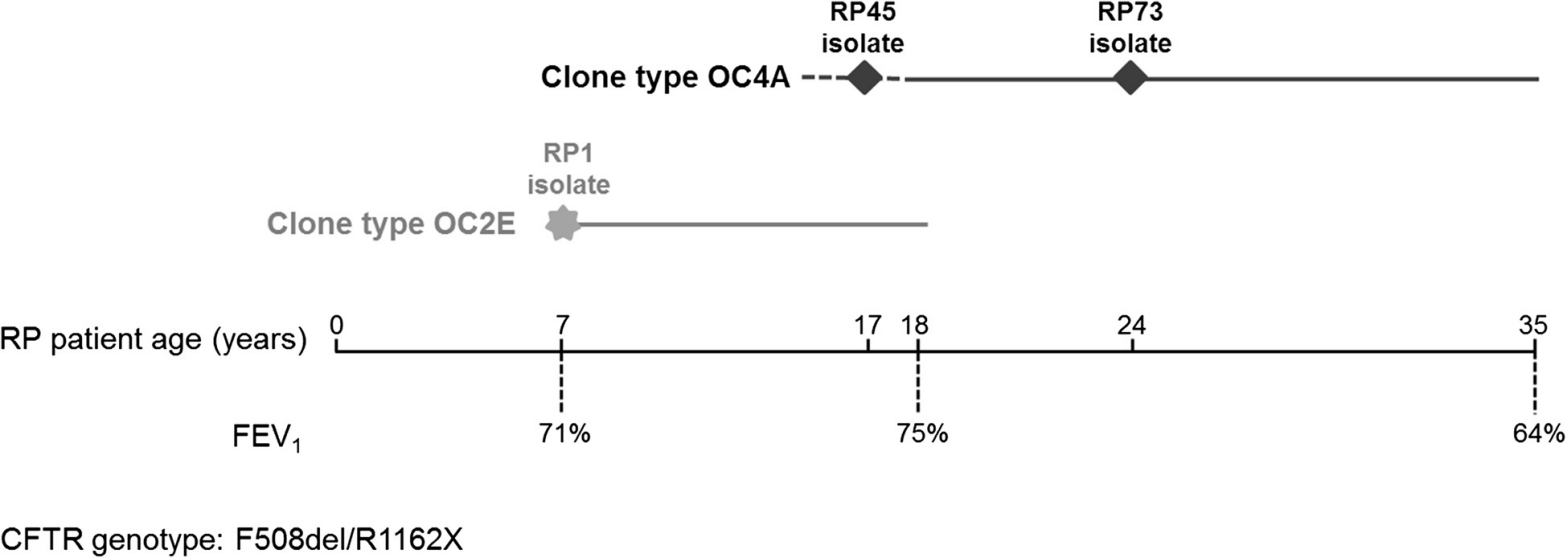
*P. aeruginosa* sequential isolates from patient RP. Two clone types (OC2E and OC4A) of *P. aeruginosa* strains were isolated from patient RP who is heterozygous for F508del and R1162X mutations in the *CFTR* gene. OC2E was isolated at the onset of chronic colonization for the first eleven years. Thereafter OC4A became the dominant clone. Strain RP1 belongs to the clone type OC2E and was the first *P .aeruginosa* strains isolated. Strains RP45 and RP7 belong to the clone type OC4A and were isolated after 10 and 16.9 years respectively after the onset of chronic colonization of the patient’s airways with *P. aeruginosa* (Additional file 1 and Cramer et al. [12]). Lung function parameters at the time of *P. aeruginosa* isolation are indicated

CF was suspected in the exocrine-insufficient patient index case RP (*CFTR* genotype: F508del/R1162X) by a positive meconium test at birth and was confirmed by pathological sweat tests at the age of 4 months. The RP patient’s airways became colonized with *P. aeruginosa* by the age of 7 years (Fig. 1). The CF clinic in Hannover has collected sequential isolates from this patient since the onset of colonization for up to 28 years [11]. The patient was chronically carrying *P. aeruginosa* isolates of clone type OC2E, for the first eleven years. During this time period strains of the clone type OC4A were sporadically isolated, but thereafter OC4A has become the dominant clone type until today. The RP patient received one to four annual 2-week courses of intravenous (iv) antipseudomonal chemotherapy since onset of colonization and was administered aerosolized colistin on a daily basis during the last 17 years. The last clone type OC2E strain was isolated from the patient’s sputum four months after the start of colistin inhalation. The patient’s clinical status remained stable during the 28 years of chronic airway infection. Lung function parameters fluctuated between 70 and 90 % predicted for forced vital capacity (FVC) and 60–80 % forced expiratory volume (FEV_1_) during the last 20 years with no tendency to irreversible decline.

In this study three *P. aeruginosa* isolates from RP patient were selected for genetic and biological characterization. RP1 was the first *P. aeruginosa* isolate and belongs to clone type OC2E, while RP45 and RP73, isolated after 10 and 16.9 years from the onset of colonization, belong to clone type OC4A (Additional file 1 and Cramer et al. [12]). Thus, RP73 *P. aeruginosa* isolate was able to establish long-term infection replacing the initially RP1 acquired isolate and likely adapting within CF airways respect to RP45.

### Pathogenicity of P. aeruginosa RP isolates in murine model of airways infection

**Fig. 2:**
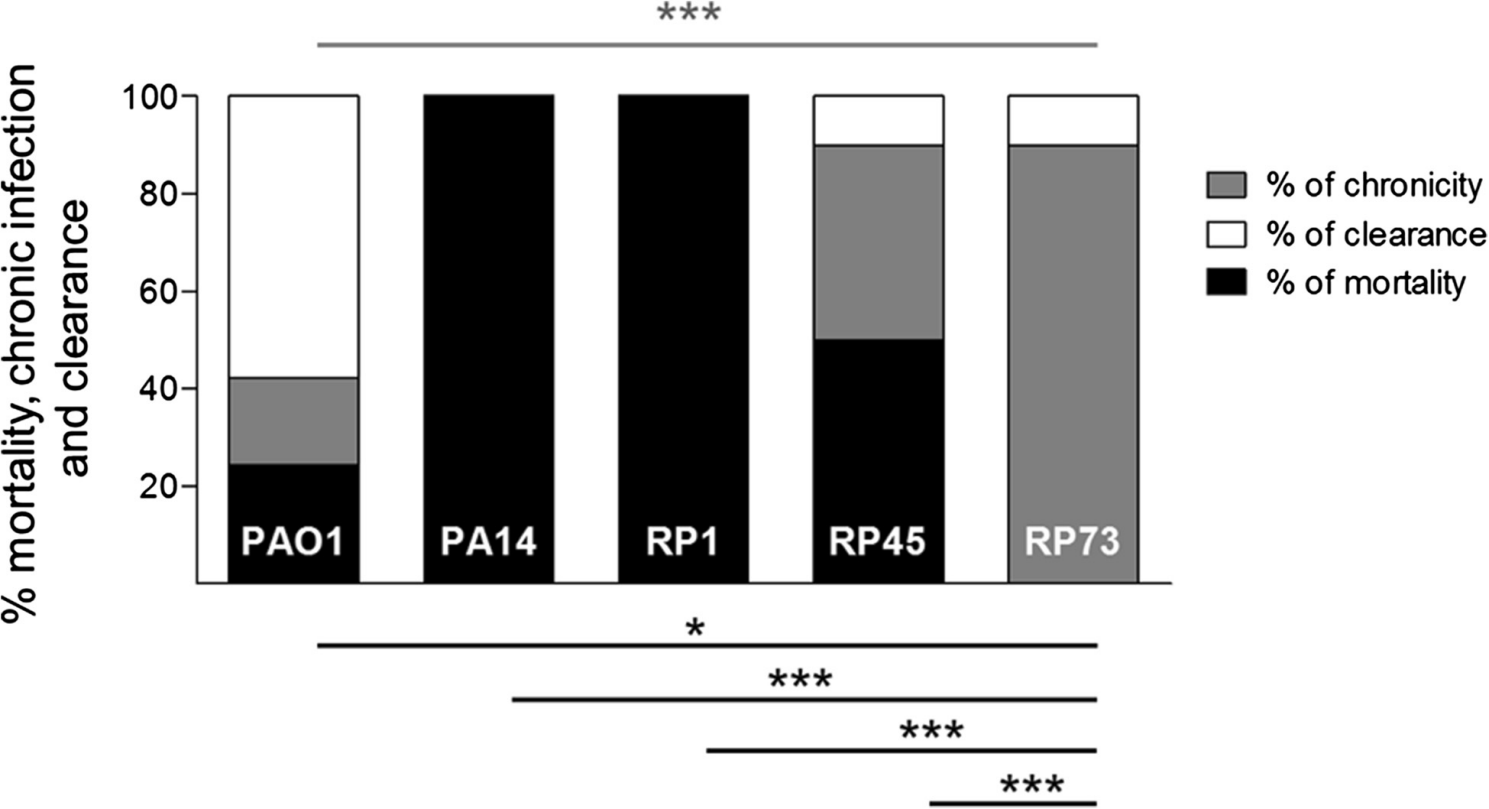
Virulence of *P. aeruginosa* RP isolates in comparison with prototype strains in murine model. C57Bl/6NCrlBR mice were infected with 1-2*10^6^ CFU/lung *P. aeruginosa* RP1, RP45, RP73, PAO1 and PA14 strains embedded in agar beads. Mortality induced by bacteremia (black) and survival (light gray) was evaluated on challenged mice. Clearance (white) and capacity to establish chronic infection (dark gray) were determined on surviving mice after 14 days. The data show the percentage of mice infected with single *P. aeruginosa* strains analyzed in two to three independent experiments. Statistical significance by Chi-square test is indicated: *P < 0.05, **P < 0.01, ***P < 0.001

To translate data from CF patients into disease models, *P. aeruginosa* clinical isolates RP1, RP45 and RP73 were tested in the agar beads mouse model of chronic airways infection in comparison to prototype PAO1 and PA14 strains [13, 14]. Bacteria embedded in the immobilizing agents appear to grow in the microaerobic/anaerobic conditions in form of microcolonies, similarly to the growth in the mucus of patients with CF [15, 16]. RP1 isolate, as well as prototype PA14 strain, caused death in all mice (100 %) within the first three days of *P. aeruginosa* infection (Fig. 2 and Additional file 2). Lower incidence of mortality (50 %) was recorded after infection with RP45 strain, while RP73 were not lethal (0 %). Thus, despite their clonality, RP45 and RP73 were significantly different in the risk of death. As previously reported, prototype PAO1 showed 24 % of acute mortality [17]. Next, the capacity to establish chronic infection in the surviving mice was assessed at 14 days. Nearly all mice had chronic airways colonization by RP45 (80 %) and RP73 (90 %), demonstrating the persistent lifestyle of this lineage among surviving mice. PAO1 strain showed less capacity to establish chronic infection (24 %). The ability of the clinical isolate RP73 to achieve long-term chronic infection associated with no risk of mortality in mice was superior to all other *P. aeruginosa* clinical strains tested in previous studies [17].

**Fig. 3:**
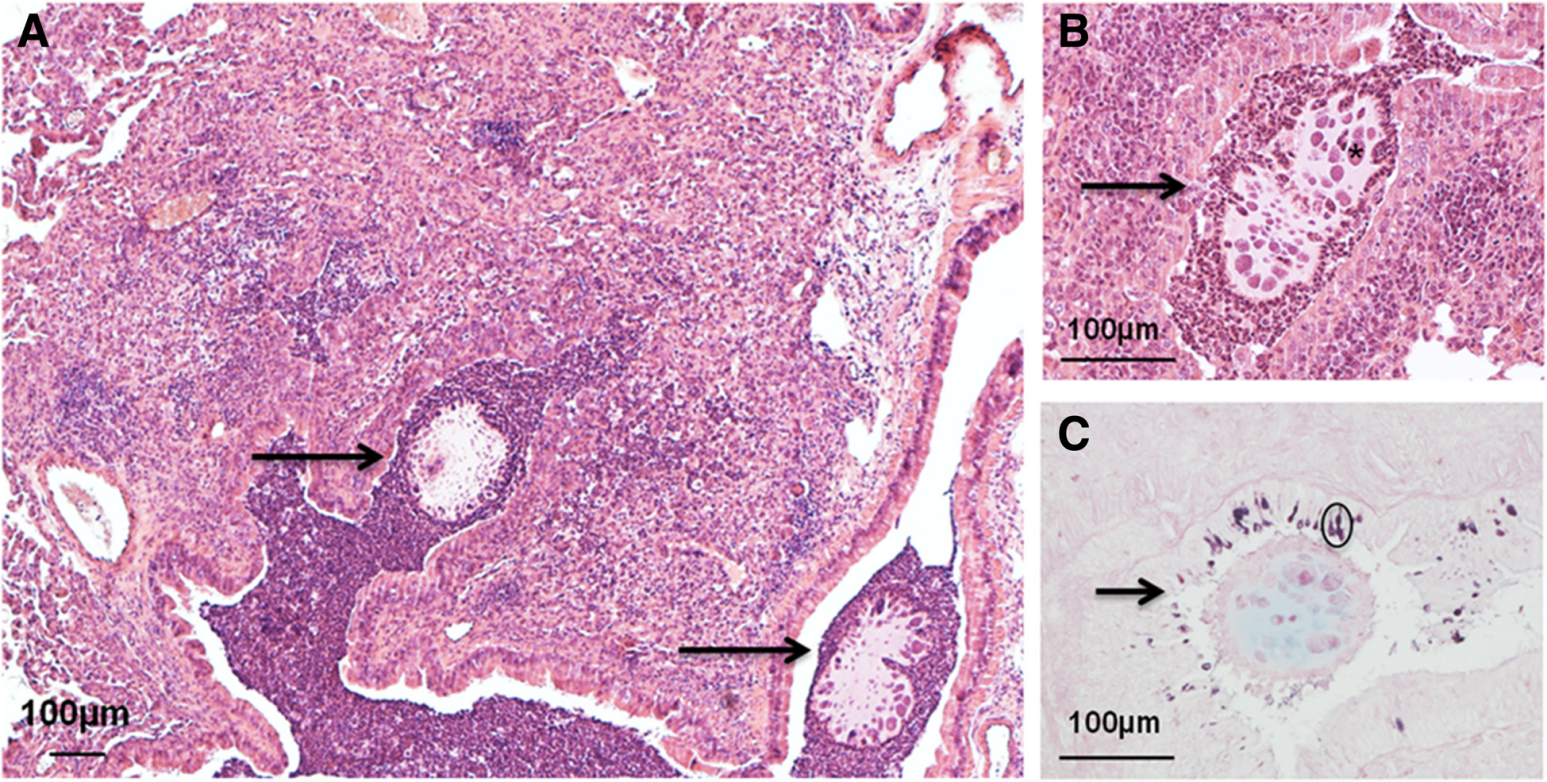
Histological lesions after chronic *P. aeruginosa* infection in mice. Mice were infected with 2x10^6^ cfu/lung of *P. aeruginosa* RP73 strain embedded in agar beads and lung harvested after 14 days from challenge. Histopathological analysis of lungs chronically infected by RP73 strain are characterised by acute and chronic lesions; the pulmonary parenchyma is infiltrated by macrophages, lymphocytes and some neutrophils (**a**). Agar beads (arrow) containing bacteria macrocolonies (*) are localised in the bronchia and surrounded by a massive neutrophils inflammation (**b**). Alcian blue staining shows mucus secretory cells hyperplasia (circle) (**c**)

To assess clinical trait of chronic infection, lung histopathology was performed after 14 days from *P. aeruginosa* challenge with the persistent RP73 isolate. Chronic pulmonary disease, including inflammation and mucus secretory cells, was detected. The bronchi were filled by a massive neutrophil inflammation, whereas the parenchyma was infiltrated by macrophages, lymphocytes and some neutrophils (Fig. 3a). Agar beads were observed in bronchial lumina (Fig. 3b). Mucous secretory cells hyperplasia (Fig. 3c) was found. These features resembled lesions found in CF patients with advanced chronic pulmonary disease [18].

### Genome sequences of RP isolates and comparative genomics analyses

**Fig. 4:**
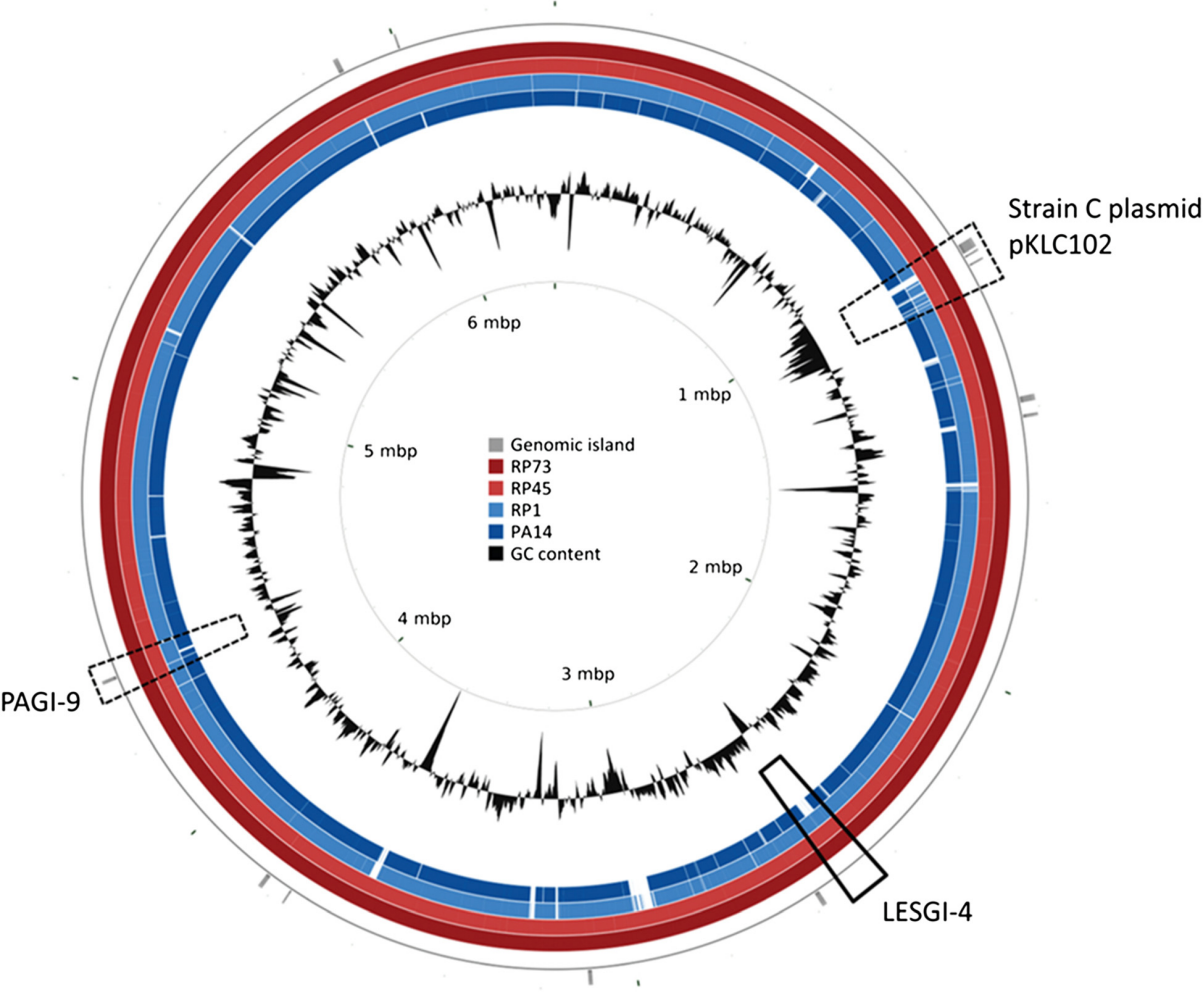
Circular map of *P. aeruginosa* RP isolates and prototype strain PA14. Circular map constructed with the CGView Comparison Tool [56]. Starting from the outside: genomic islands predicted with IslandViewer (see Table 1 for details) [52], RP73, RP45, RP1, PA14 and GC content. Colored regions are shared with RP73 according to blast search. Dotted lines: known genomic islands (GIs) that distinguish RP73 because they are incomplete or absent in the 12 complete *P. aeruginosa* genomes available at pseudomonas.com. RP isolates also carry LESGI-4, identified in the Liverpool epidemic strain

To link the persistent lifestyle with a genetic basis, we sequenced the genome of RP73 [19], in addition to those of preceding RP1 and clonal RP45 isolates, and performed comparative genomic analysis. The fully assembled RP73 genome consists of a single circular chromosome of 6,342,034 base pairs (Additional file 3 for genome description). Twelve genomic islands were predicted in this genome (Table 1); three of them distinguished RP73 from other prototype strains and corresponded to regions of genome plasticity (Fig. 4) [5]. They include known genomic islands PAGI-9, which is similar to rearrangement hot spots (*Rhs*) [20], and plasmid pKLC102, which carries the *pil* gene cluster and *chvB* glucan synthetase [21]. Nucleotide blast search on NCBI limited to *P. aeruginosa* showed that the former can be found in multiple clinical isolates, while the latter is identical to RP73 only in strain 8380, isolated from the human gut. However, plasmid pKLC102 is often partially present [22]. A SMC4389 CRISPR repeat sequence also differentiates RP73 from most prototype strains [6]. In fact, blast search for this sequence resulted in a single hit from soil strain *Azotobacter chroococcum* NCIMB 8003. The RP73 genome also contains full length LESGI-4, which was identified in the Liverpool epidemic strain (LES) [23]. Genomic islands predicted in RP73 were investigated in the draft genomes of RP1 and RP45. While RP45 carries all 12, RP1 lacks full-length plasmid pKLC102 and an ABC transporter protein. A circular map comparing the 3 sequenced RP genomes clearly shows the genomic similarity between RP45 and RP73 on one hand, and between RP1 and strain PA14, which showed similar results in the murine infection model, on the other (Fig. 4).

**Table 1:** Predicted genomic islands in the genome of *P. aeruginosa* RP73 and comparison with other RP isolates and prototype strains

RP73 genomic island annotation						Other strains from patient RP		Prototype strains			
Predicted Island	Start position	End position	Size (bp)	Annotation	Region of genome plasticity	RP45	RP1	PA14	PAO1	LESB58	PA7
1–3	1023578	1126198	102620	*Pseudomonas aeruginosa* strain C plasmid pKLC102	41	P	*	*	-	-	*
4	1372342	1385723	13381	Major facilitator transporter, 2-isopropylmalate synthase, putative membrane-bound lytic murein transglycolase A, hypothetical proteins	na	P	P	P	P	P	-
5	1408824	1421034	12210	*Pseudomonas aeruginosa* strain SMC4389 CRISPR repeat sequence	12	P	P	-	-	-	-
6	2576892	2587628	10736	Non-ribosomal peptide synthetases, hypothetical proteins	na	P	P	P	P	P	-
7	3092401	3101220	8819	Type II secretion system proteins, ABC transporter permease, hypothetical proteins	na	P	P	P	P	P	-
8	3764047	3768307	4260	Hypothetical proteins	na	P	P	P	P	P	-
9	3818768	3828628	9860	Non-ribosomal peptide synthetase, FAD-dependent monooxygenase, short chain dehydrogenase, cytochrome P450, 3-oxoacyl-(acyl carrier protein) synthase III, acyl carrier protein, major facilitator transporter	na	P	P	P	P	P	-
10	4356197	4363888	7691	*Pseudomonas aeruginosa* PAGI-9 genomic island sequence	89	P	P	-	-	-	-
11	5866390	5877731	11341	Putative short-chain dehydrogenase, ABC transporter ATP-binding protein, hypothetical proteins	na	P	*	*	*	*	*
12	6002572	6007043	4471	Hypothetical proteins	na	P	P	-	P	P	-

Genomic islands were predicted using Island Viewer [52] and described based on annotation with xBase [53] using *P. aeruginosa* PAO1 as a reference genome

Colocalization with regions of genome plasticity previously described by Klockgether et al. [5], *P* present, *: partially present (20–90 % coverage), -: absent

**Fig. 5:**
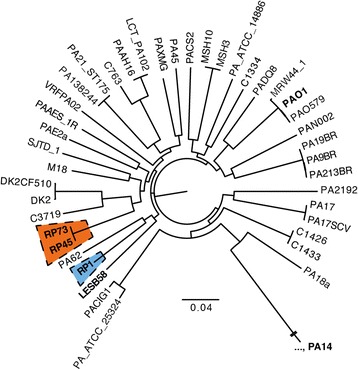
Core genome phylogeny for RP isolates and strains representative of *P. aeruginosa* diversity. The figure represents a partial view of the tree to show the relationships between RP1, RP45 and RP73. The position of RP1 is indicated in blue, while the position of RP45 and RP73 is indicated in red. PA14 is distantly related to all these strains

In order to study the evolution of chronic infection and determine which genetic determinants are involved in this process, further comparisons were made among RP isolates. First, analyses were carried out to study the relationship between these three strains. A core genome phylogeny was performed using 53 sequences from a previous study [24] representing an extensive sampling of *P. aeruginosa*’s diversity. In the resulting tree (Fig. 5), RP73 and RP45 cluster together while RP1 is found in a different and independent branch. A multi-locus sequence typing (MLST) analysis was also performed. RP73 and RP45 shared the same MLST profile while RP1 showed a different one (Additional file 4). All these analyses suggest that RP73 and RP45 are close to each other from a genetic point of view, while RP1 is more distantly related.

In order to explain the molecular bases of the development of chronic infection in a CF context, SNPs were determined between PA14 and RP1 compared to RP45 and RP73. A genomic comparison between PAO1 and RP1 was already performed by Hilker et al. [22]. A total of 54,621 SNPs was found by taking PA14 as reference strain and searching for positions at which at least one among RP1, RP45 and RP73 strains showed a different nucleotide compared to PA14. About one fifth of these SNPs (10,819) were non-synonymous substitutions (Additional file 5) that are likely to have an effect on protein function and structure. Several of these SNPs were located in virulence genes representing good candidates to explain the diversity in patterns of mortality and chronicity we observed.

### Virulence factors of RP isolates and their putative role in pathogenicity

The genome of RP isolates contains most of the virulence factors, described for other *P. aeruginosa* genomes and identified in the Virulence Factor (VF) database (http://www.mgc.ac.cn/VFs/) [25], with few exceptions. Several VFs of RP73 isolate shows signatures of pathoadaptive mutations within the genome when compared to the preceding clonal RP45 isolate as reported in Additional files 5 and 6. Phenotypic characterization of RP isolates is reported (Table 2) and their putative role in pathogenicity discussed.

**Table 2:** Phenotypic characterization of *P. aeruginosa* RP isolates and prototype strains

Strain	Mucoidy	Twitching^*^ (ø cm)	Swarming^*^ (ø cm)	Protease (ø cm)	Siderophore (ø cm)	LasR henotype^§^	Pyocyanin ^#^	Biofilm°	Antibiotic resistance
RP1	-	2.3	3.4	2.1	1.2	-	0.094 ± 0.016	0.332 ± 0.141	-
RP45	-	-	-	-	-	+	0.051 ± 0.015	0.493 ± 0.146	GEN
RP73	-	-	-	-	1.3	+	0.05 ± 0.013	1.289 ± 0.596	AMK; CAZ; GEN; IMP; MER
PAO1	-	1.2	2.6	1.8	2.0	-	0.127 ± 0.015	1.719 ± 0.217	-
PA14	-	1.2	5	2.1	2.0	-	0.09 ± 0	3.553 ± 0.457	-

*Indicates twitching and swarming motility zone diameter, as measured by subsurface stab assay

§Isolates with iridescent and metallic sheen of the colony surface, that is typical for a *lasR* mutant, are indicated (+)

#Indicates mean value ± SD at 26 h. Values ≤ 0.05 indicate no production of pyocyanin

°Indicates mean value ± SD at 24 h

*AMK* amikacin, *CAZ* ceftazidime, *GEN* gentamicin, *IMP* imipenem, *MER* meropenem

#### Motility, adherence and cell interaction

Pili, flagella and outer membrane proteins promote motility, attach to epithelial or endothelial cells, activate or inactivate host cellular pathways and immune responses [26]. These *P. aeruginosa* VFs play a key role in acute infection and are present in RP1 isolate. Variations in the twitching and swimming motility are common in *P. aeruginosa* isolates from CF patients and described to be hallmarks of bacterial adaptation to the airways [7, 27]. Both RP73 and its clonal RP45 isolate did not encode *pilV*, *pilW*, *pilY2* and *fimT* and carried a premature stop mutation in *pilO* (Table 3); while *pilA* and *pilC* were deleted in all isolates from RP patient. The RP73 and RP45 phenotypes are consistent with the absence of twitching and swimming motility (Table 2). Lack of motility was associated with decreased virulence in models of acute infection [28, 29] and increased risk of chronic infection [17]. Our results obtained in RP73 and RP45-infected mice (Fig. 2) are consistent with the observation that unlike strains RP1, PA14 and PAO1, favor long-term persistence.

**Table 3:** Comparative pathogenomics of mutations present in the major virulence factors of RP isolates respect to the prototype strain PAO1 and evaluation in others *P. aeruginosa* sequenced strains

Virulence factors	ORFs	PAO1^1^	PA14^1^	LESB58^1^	PA7^1^	RP1^2^	RP45^2^	Mutation in RP45	RP73^2^	Mutation in RP73
Type IV Pili biosynthesis	*pilA*	PA4525	PA14_58730	PLES_49071	PSPA7_5161	deleted^3^	deleted^3^		deleted	
	*pilC*	PA4527	PA14_58760	PLES_49101	PSPA7_5163	deleted^3^	deleted^3^		deleted	
	*pilO*	PA5042	PA14_66640	PLES_54321	PSPA7_5779	present	present	stop codon after 138 aa	present	stop codon after 138 aa
	*pilV*	PA4551	deleted	PLES_49341	PSPA7_5191	present	deleted^4^		deleted	
	*pilW*	PA4552	PA14_60290	PLES_49351	PSPA7_5192	present	deleted^4^		deleted	
	*pilY2*	PA4555	deleted	PLES_49381	PSPA7_5195	present	deleted^4^		deleted	
	*fimT*	PA4549	deleted	PLES_49321	PSPA7_5189	present	deleted^3^		deleted	
Alginate regulation	*algP/algR3*	PA5253	PA14_69370	PLES_56471	PSPA7_5998	present	present		present	
Pyochelin	*pchD*	PA4228	PA14_09240	PLES_06991	PSPA7_0872	present	present	stop codon after 276 aa	present	stop codon after 276 aa
Pyoverdin	*pvdD*	PA2399	PA14_33650	PLES_28971	deleted	present^5^	present		present	stop codon after 839 aa
Phospholipase D	*pldA*	PA3487	PA14_18970	deleted	deleted	deleted^4^	deleted^4^		deleted	
N-(3-oxo-dodecanoyl)-L-homoserine lactone QS system	*lasR*	PA1430	PA14_45960	PLES_39841	PSPA7_3898	present	present	stop codon after 220 aa	present	stop codon after 220 aa
Type III Secretion System	*pscQ*	PA1694	PA14_42610	PLES_36321	deleted	present	present		deleted	
	*pscP*	PA1695	PA14_42600	deleted	deleted	present	present^5^		deleted	
	*pscO*	PA1696	PA14_42580	PLES_36311	deleted	present	present		deleted	
	*pscN*	PA1697	PA14_42570	PLES_36301	deleted	present	present		deleted	
	*poPN*	PA1698	PA14_42550	PLES_36291	deleted	present	present		deleted	
	*pcr1*	PA1699	PA14_42540	PLES_36281	deleted	present	present		deleted	
	*pcr2*	PA1700	PA14_42530	PLES_36271	deleted	present	present		deleted	
	*pcr3*	PA1701	PA14_42520	PLES_36261	deleted	present	present		deleted	
	*pcr4*	PA1702	PA14_42510	PLES_36251	deleted	present	present		deleted	
	*pcrD*	PA1703	PA14_42500	PLES_36241	deleted	present	present		deleted	
	*pcrR*	PA1704	PA14_42490	PLES_36231	deleted	present	present		deleted	
	*pcrG*	PA1705	PA14_42480	PLES_36221	deleted	present	present		deleted	
	*pcrV*	PA1706	PA14_42470	PLES_36211	deleted	present	present		deleted	
	*pcrH*	PA1707	PA14_42460	deleted	deleted	present	present		deleted	
	*poPB*	PA1708	PA14_42450	PLES_36201	deleted	present	present		deleted	
	*poPD*	PA1709	PA14_42440	PLES_36191	deleted	present	present		deleted	
	*exsC*	PA1710	PA14_42430	PLES_36181	deleted	present	present		deleted	
	*exsE*	PA1711	PA14_42410	deleted	deleted	present	present		deleted	
	*exsB*	PA1712	PA14_42400	PLES_36171	deleted	present	present		deleted	
	*exsA*	PA1713	PA14_42390	PLES_36161	deleted	present	present		deleted	
	*exsD*	PA1714	PA14_42380	PLES_36151	deleted	present	present		deleted	
	*pscB*	PA1715	PA14_42360	PLES_36141	deleted	present	present		deleted	
	*pscC*	PA1716	PA14_42350	PLES_36131	deleted	present	present		deleted	
	*pscD*	PA1717	PA14_42340	PLES_36121	deleted	present^5^	present		deleted	
	*pscE*	PA1718	PA14_42320	PLES_36111	deleted	present	present		deleted	
	*pscF*	PA1719	PA14_42310	PLES_36101	deleted	present	present		deleted	
	*pscG*	PA1720	PA14_42300	PLES_36091	deleted	present	present		deleted	
	*pscH*	PA1721	PA14_42290	PLES_36081	deleted	present	present		deleted	
	*pscI*	PA1722	PA14_42280	PLES_36071	deleted	present	present		deleted	
	*pscJ*	PA1723	PA14_42270	PLES_36061	deleted	present	present		deleted	
	*pscK*	PA1724	PA14_42260	PLES_36051	deleted	present	present		deleted	
	*pscL*	PA1725	PA14_42250	PLES_36041	deleted	present	present		deleted	
LPS	*lpxO2*	PA0936	PA14_52150	PLES_43801	PSPA7_4774	present	present		present	stop codon after 145 aa
Efflux pumps	*mexA*	PA0425	PA14_05530	PLES_04231	PSPA7_0525	present	present	+29 aa at N-ter	present	+29 aa at N-ter
	*mexT*	PA2492	PA14_32410	pseudogene	PSPA7_2746	present	present	N-ter not conserved	present	N-ter not conserved
Antibiotic resistance	*arr*	PA2818	deleted	deleted	PSPA7_2339	deleted^3^	deleted^3^		deleted	

^1^Sources for PAO1, PA14, LESB58 and PA7 information are the Virulence Factor Database

(http://www.mgc.ac.cn/VFs/) and Pseudomonas Database (www.pseudomonas.com)

^2^Identified by local blast (>90 % query coverage, >90 % sequence similarity) using the PAO1 gene sequences as query

^3^Presence of at least one flanking region (500 bp) confirmed by blast search on contigs; gene not located at contig edge

^4^Flanking regions not found, but absence of the gene is consistent with a deletion in isolate RP73

^5^Gene is present, but separated onto multiple contigs

Lipopolysaccharides (LPSs) are potent immune stimulants through their interactions with Toll-like receptor 4 (TLR4). *P. aeruginosa* strains isolated from CF patients evolved the capacity to reduce host immuno-detection by modulating LPS structure [30]. Biochemical and biological characterization of RP73 LPS showed to possess an under-acylated lipid A leading to a lower pro-inflammatory capacity in a murine model of intranasal instillation when compared to the LPS from the prototype strain PAO1 [31]. This structure is distinguished by the absence of hexa- or hepta-acylated lipid A species that are typical phenotypic changes that can occur on the LPS molecule of a *P. aeruginosa* chronic strain. Furthermore, RP73 carries an R-type LPS without O-antigen in which the lipid A is covalently linked to the core oligosaccharide region. Absence of LPS O-antigen in RP73 suggests an adaptation of this strain to persistent lifestyle.

Among genes responsible for lipid A modification (*lpxO1, lpxO2, phoP, phoQ, pagL* and *oprH*) [30], our genetic characterization showed that RP73 isolate carries a stop mutation and lacks of the C-terminus of the protein in *lpxO2* compared to the preceding clonal RP45, as well as RP1 isolate and prototype PAO1 and PA14 strains (Table 3). Both RP45 and RP73 isolates carry a non-synonimous SNP in the sequence of pagL compared with RP1 and PA14 (Additional files 5 and 6). These data suggests that an adaptive process has occurred to the LPS structure of the RP lineage in the period of time between the isolation of RP45 and RP73.

#### Secretion systems and toxins

In *P. aeruginosa* genome, the genes (*psc, pcr, exs* and *pop*) encoding the type III secretion system (T3SS) are clustered together. The *psc* and *prc* genes primarily encode components of the bacterial secretion apparatus whereas the *exs* gene products are involved in regulation of TTSS. Two *pop* genes encode proteins (PopB and PopD) essential for the translocation of the effectors into host cells. Remarkably, RP73 isolate lost the entire 32-gene cluster encoding the T3SS which was present in the preceding clonal RP45, as well as RP1 and prototype PAO1 and PA14 strains (Table 3) [25]. However, the *exoS, exoT*, and *exoY* genes encoding for the “T3SS translocated effectors” are still present both in RP73 and RP45 genome with two non-synonymous SNPs recorded in the *exoT* when compared with RP1 and PA14 (Table 3 and Additional files 5 and 6). T3SS is an important virulence determinant of *P. aeruginosa* which may act at the site of infection and contribute to subversion of the host immune response. In contrast to acute infection, small proportion of isolates infecting CF patients secrete T3SS proteins and this proportion decreases with duration of infection [32]. We speculate that the differences in the risk of mortality associated to RP73 and RP45 isolates may be linked to absence or presence of the T3SS that changed during the progression of CF chronic infection.

Genes encoding for *toxA, hcnA, hcnB* and *hcnC* are present. However, RP73 has two non-synonymous SNPs compared with its clonal isolate RP45, as well as RP1 and PA14, in *hcnC* gene. If we consider the exotoxin A, we observed several non-synonymous SNPs (Table 3 and Additional file 5).

Finally, *pldA* coding for the periplasmic phospholipase D (Table 3), one of effectors of the type VI secretion system [33], is absent in RP73 genome. However *pldA* is reported to be not conserved among *P. aeruginosa* genomes [34].

#### Iron uptake and pigment

The ability to produce siderophores, like pyochelin and pyoverdine, has been linked to the bacterial pathogenic potential. Phenotypic assay showed that RP isolates secreted lower (RP73 and RP1) or no (RP45) levels of siderophores when compared to PAO1 and PA14 (Table 2). When we look at the genome sequence, both RP73 and RP45 carries stop mutations both in *pchD,* while PvdD protein is interrupted in RP73 only. PchD and PvdD are both involved in the synthesis of the two principal siderophores (Table 3). PvdD production was shown to be required for airways bacterial colonization in rat, lethal virulence in burned and immunosuppressed mouse models [35, 36]. Pyoverdine was detected in the sputa of CF patients [37], while in a larger study, one-third of sputa positive for *P. aeruginosa* contained no detectable pyoverdine [38]. These data suggests that pyoverdine-mediated iron uptake may not always be essential for chronic infection and other mechanisms are active in CF [39].

Phenotypic tests showed that RP73 and RP45 are able to secrete less pyocyanin when compared to RP1, PAO1 and PA14 strains (Table 2). No changes in genes involved in pyocyanin biosynthesis were found, however in RP73 and RP45 were found non-synonymous SNPs in several genes belonging to the two phenazine biosynthesis operons (*phzA1B1C1D1E1F1G1* and *phzA2B2C2D2E2F2G2)* if compared with the non-clonal isolate RP1 and the prototype strain PA14 (Additional file 5). Pyocyanin is required for full virulence in animal models and has been detected in patients’ airway secretions, promoting virulence by interfering with several cellular functions in host cells including electron transport, cellular respiration, energy metabolism, gene expression, and innate immune mechanisms [40].

#### Mucoidy

RP isolates are phenotypically non-mucoid (Table 2) with absence of mutations in *mucABD* locus. Other key regulators of alginate pathway are present (Table 3). The phenotypic switch to mucoidy in *P. aeruginosa* infections is a well-established paradigm in CF. Infection with the mucoid phenotype, which produces large amounts of the exopolysaccharide alginate, has been associated with a more rapid decline in pulmonary function than infection with non-mucoid bacteria [41]. However, some but not all *P. aeruginosa* isolates became mucoid in the CF lung suggesting that a mucoid phenotype did not always confer a selective advantage to bacterial cells in persistence [42]. In mouse model, CF clonal strains, displaying a mucoid and a non-mucoid phenotype, showed a similar capacity of persistence [17]. Our data obtained in mouse model with RP73 and RP45 isolates support the notion that non-mucoid *P. aeruginosa* strains are able of long-term persistence.

#### Quorum sensing

In *P. aeruginosa*, many virulence determinants and secondary metabolites are regulated in a cell population density-dependent manner via cell-to-cell communication or “quorum sensing” (QS) [43]. *P. aeruginosa* possesses two *N-*acylhomoserine lactone (AHL)-dependent QS systems. These are termed the *las* and *rhl* systems, consisting of the LuxRI homologues, LasRI and RhlRI, respectively. RP isolates have no mutations in *lasI*, *rhlR* and *rhlI* genes. However, inactivation of the transcriptional regulator LasR, carrying a stop mutation in the gene sequence (Table 3), was found both in RP73 and RP45, while the RP1 has no changes. The distinctive *lasR* mutant phenotype was confirmed by colony morphology that includes surface iridescent sheen and colony flattening exclusively in RP73 and RP45 (Table 2) [44].

Mutations in *lasR* lead to several phenotypic changes of potential clinical significance, including a growth advantage in amino acid abundant CF airway secretions. LasR regulates the production of virulence factors (elastase, protease, alkaline protease and exotoxin A) affecting the immune response and antibiotic resistance [45]. Most importantly, *lasR* mutations are often associated with the progression of CF lung disease and may serve as a marker of early CF adaptive change of prognostic significance [46].

#### Antibiotic resistance

RP73 showed remarkable resistance to most of the antibiotic classes while the preceding RP45 and RP1 isolates were not, indicating an increased treatment-refractory during the course of the chronic infection in this CF patient (Table 2). A strong link between antibiotic resistance and hypermutation was observed in patients with CF [47]. However, RP73 strain does not have mutations in *mutS*, *mutL* and *uvrD,* described previously as responsible for the hypermutable phenotype [48]. Regarding efflux pumps, the RP73 strain did not show mutation in *mexEF-oprN*, *mexCD-oprJ* and *mexXY*. Respectively one and six non-synonymous SNPs are present in the sequence of *mexC* and *mexD* of RP73 when compared with its clonal isolate RP45. No insertions or deletions in *ampC*, *ampR*, *mexR*, *mexZ* and *oprD* were detected. An insertion at the N-terminal of MexA and a non-synonymous SNP was found in RP73 and RP45 (Table 3 and Additional file 5). MexA belongs to the efflux pump complex MexAB-OprM, which is anchored to the inner membrane via N-terminal fatty acids. Adaptive mutations in *mexA* have been reported in CF isolates [7].

Additional modifications were detected at the N-terminal of *mexT*, which is not conserved in RP73 and RP45 (Table 3). An additional non-synonymous aa change at position 128 was found in RP73 when compared to RP45 (Additional file 5). MexT plays a pleiotropic role in modulating *P. aeruginosa* virulence such as TTSS, pyocyanin production and early surface attachment [49]. Similarly to MexA, also MexT is an hallmark of *P. aeruginosa* adaptation in CF patients [7]. Among the additional 58 PAO1 coding sequence annotated as “antibiotic resistance and susceptibility”, only *arr*, a putative aminoglycoside response regulator, is absent in all strains from RP patient.

## Conclusions

Taken together, our study combined clinical data, whole-genome analysis and animal models to link the persistent lifestyle of *P. aeruginosa* in CF lungs with the bacterial genetic basis. Starting from a clinical case of CF, *P. aeruginosa* RP73 was isolated after long-term chronic infection and compared with the preceding RP1 and clonal RP45, as well as prototype PAO1 and PA14 strains. When tested in the animal model, *P. aeruginosa* RP73 isolate, but not other strains, mimics most of the traits of airways infection and inflammation observed in CF patients. These results suggested that key features of RP73 isolate may contribute to its pathogenesis. The genome sequence of RP73 and comparative genomics analysis with other *P. aeruginosa* genomes, pointed clearly to signatures of pathoadaptive mutations within the genome. This in turn correlated with the major impact on the in vitro phenotypes and in vivo maintenance observed and described here. Our findings support and better define the hypothesis that genes encoding major virulence factors are deleted and/or contain beneficial mutations when *P. aeruginosa* establishes long-term chronic infection. The results presented in this study provide important information with respect to the *P. aeruginosa* mosaic genome structure and chronic infections found in CF patients.

## Methods

### Ethics statement

Animal studies adhered strictly to the Italian Ministry of Health guidelines for the use and care of experimental animals. The use of the clinical data is in line with study no. 3739 which has been approved by the Ethics Commission of Hannover Medical School. The patient and her parents provided informed consent prior to sampling of strains and storage of clinical data.

### Bacterial strains and CF patient

CF clinical *P. aeruginosa* RP1, RP45 and RP73 isolates were chosen from the collection of the CF clinic Medizinische Hochschule Hannover, Germany. Genotypic analysis by multimarker array and phenotypic data of *P. aeruginosa* strains were partly published [50]. *P. aeruginosa* was cultured in *Pseudomonas* isolation agar (PIA) or Trypticase Soy Broth (TSB) at 37 °C. CF patient gave informed consent before the sample collection. Approval for storing of biological materials was obtained by the Hannover Medical School, Germany.

### Phenotypic characterization

Swimming and twitching capacities, protease, siderophore and pyocyanin secretion, hemolytic activity, and LasR mutant phenotypic analysis were assayed as described in the online data supplement.

### Genome sequencing

The genome of RP73 was previously published [19]. Genomic DNA from strains RP1 and RP45 was isolated from overnight cultures using the DNeasy Blood and Tissue Kit (QIAGEN). Genomic DNA (500 ng) was mechanically fragmented for 40 s using a Covaris M220 (Covaris, Woburn MA, USA) with default settings. Fragmented DNA was transferred to PCR tubes and library synthesis was performed with the Kapa Hyperprep kit (Kapa biosystems, Wilmington MA, USA) according to manufacturer’s instructions. TruSeq HT adapters (Illumina, SanDiego, CA, USA) were used to barcode the samples and each library was sequenced in 1/48 of an Illumina MiSeq 300 bp paired-end run at the Plateforme d’Analyses Génomiques of the Institut de Biologie Intégrative et des Systèmes (Laval University, Quebec, Canada). Sequencing data for each genome was assembled with the A5 pipeline [51]. Whole genome shotgun projects has been deposited at DDBJ/EMBL/GenBank under accessions LNBU00000000 (RP1) and LNDM00000000 (RP45).

### Genomic analyses

Blast (NCBI) was used to compare the genome of RP73 to prototype strains and to the complete *Pseudomonas aeruginosa* content in Genbank. Genomic islands were predicted with Island Viewer [52] and annotated with xBase [53]. The core genome phylogeny was determined using the Harvest suite [51]. The data set of sequences we used to generate the core phylogeny includes 53 strains representative of P. aeruginosa diversity. MLST profiles were determined combining the results obtained from the pubmlst database (http://pubmlst.org) and the SRST2 software package [54]. SNPs between PA14 and RP1, RP45 and RP73 were detected with the Samtools software package [55] (samtools mpileup options: -C 50, SNPs with quality score of less than 30 were discarded).

### Mouse model of P. aeruginosa acute and chronic lung infection

For chronic infection, C57BL/6NCrlBR male mice (20–22 g, Charles River) were infected with 1-2×10^6^ CFUs of *P. aeruginosa* strains, embedded in agar beads [15, 17]. Fourteen days post-challenge lungs were recovered, homogenized and plated for CFUs counting. In additional group of mice, the lungs were excised for histopathology. Additional details are reported in the Online data supplement. Student’s t-test and the *χ*^2^ test considering *p* < 0.05 as the limit of statistical significance was performed.

#### Availability of supporting data

The data sets supporting the results of this article are included within the article and its additional files.

## Additional files

Additional file 1:
**PFGE of** ***P. aeruginosa*** **RP isolates.** (PNG 112 kb) Additional file 2:
**Virulence of** ***P. aeruginosa*** **RP isolates, and prototype strains in a murine model of chronic airways infection.** (DOC 29 kb) Additional file 3:
**Genomic features of RP strains compared to others complete** ***P. aeruginosa*** **genomes [** 4 **,** 12 **,** 19 **,** 23 **,** 57 **,** 58 **].** (DOC 41 kb) Additional file 4:
**MLST typing of RP isolates [** 55 **].** (DOC 28 kb) Additional file 5:
**Non-synonymous SNPs between prototype strain PA14 and strains RP73, RP45 and RP1.** (XLS 2195 kb) Additional file 6:
**Non-synonymous SNPs between prototype strain PA14 and strains RP73, RP45 and RP1 located in known virulence factors.** (XLS 31 kb)

## Abbreviations

AHL: *N-*acylhomoserine lactone
CDSs: coding sequences
CF: cystic fibrosis
CFUs: colony forming units
COPD: chronic obstructive pulmonary disease
FEV: forced expiratory volume
FVC: forced vital capacity
GIs: genomic islands
LES: liverpool epidemic strain
LPSs: lipopolysaccharides
MDR: multidrug-resistant
ORFs: open reading frames
PIA: *Pseudomonas* isolation agar
PFGE: pulsed field gel electrophoresis
TLR4: toll-like receptor 4
TSB: trypticase soy broth
T3SS: type III secretion system
VF: virulence factor
