# Supplementary figures and images for: Comparative genomics and biological characterization of sequential Pseudomonas aeruginosa isolates from persistent airways infection
Source: BMC Genomics. 2015 Dec 29;16:1105. doi: 10.1186/s12864-015-2276-8 (PMC4696338; doi:10.1186/s12864-015-2276-8)

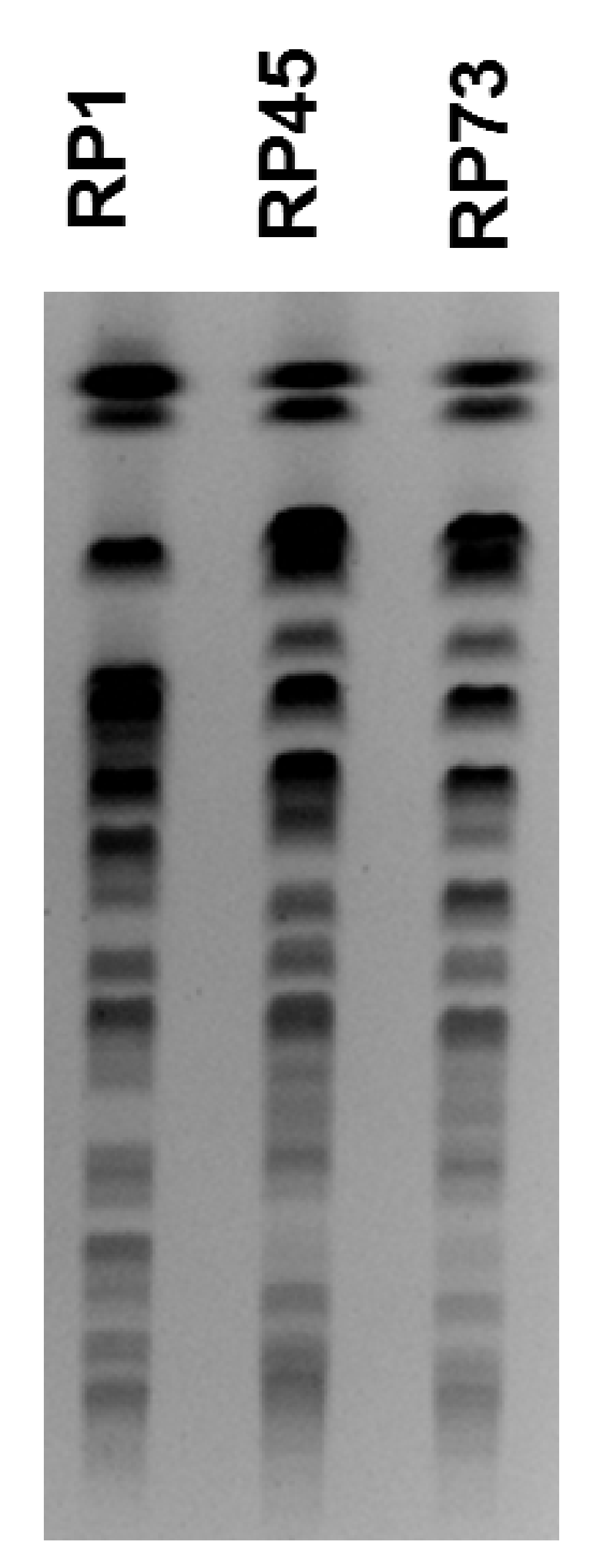

Supplement: Additional file 1: — PFGE of P. aeruginosa RP isolates. (PNG 112 kb) [file 12864_2015_2276_MOESM1_ESM.png]
