# Supplementary material for: Comparative genomics and biological characterization of sequential Pseudomonas aeruginosa isolates from persistent airways infection
Source: BMC Genomics. 2015 Dec 29;16:1105. doi: 10.1186/s12864-015-2276-8 (PMC4696338; doi:10.1186/s12864-015-2276-8)
Supplement: Additional file 2: — Virulence of P. aeruginosa RP isolates, and prototype strains in a murine model of chronic airways infection. (DOC 29 kb) [file 12864_2015_2276_MOESM2_ESM.doc]

**Additional File 2. Virulence of *P. aeruginosa* RP isolates, and prototype strains in a murine model of chronic airways infection*.***

| **Strain** | **No. of mice** | **Mortality %**  **(No. of dead/total mice)** | **Chronic infection % (No. of infected /surviving mice)** | **CFU/lung of surviving mice** |
| --- | --- | --- | --- | --- |
| PAO1 | 66 | 24.2 (16/66) | 24.0 (12/50) | 2.80*10^4^(12) |
| PA14 | 14 | 100 (14/14) | - | - |
| RP1 | 12 | 100 (12/12) | - | - |
| RP45 | 10 | 50 (5/10) | 80 (4/5) | 2.74*10^5^ (4) |
| RP73 | 20 | 0(0/20) | 90 (18/20) | 5.65*10^4^ (18) |
