# Supplementary material for: Comparative genomics and biological characterization of sequential Pseudomonas aeruginosa isolates from persistent airways infection
Source: BMC Genomics. 2015 Dec 29;16:1105. doi: 10.1186/s12864-015-2276-8 (PMC4696338; doi:10.1186/s12864-015-2276-8)
Supplement: Additional file 3: — Genomic features of RP strains compared to others complete P. aeruginosa genomes [ 4 , 12 , 19 , 23 , 57 , 58 ]. (DOC 41 kb) [file 12864_2015_2276_MOESM3_ESM.doc]

**Additional File 3: Genomic features of RP strains compared to others complete *P. aeruginosa* genomes.**

| **Strain** | **Source** | **Genome size**  **(Mbp)** | **GC-content (%)** | **N. of protein coding ORFs** | **Reference** |
| --- | --- | --- | --- | --- | --- |
| **RP1*** | CF-patient | 6.933 |  |  | this paper |
| **RP45*** | CF-patient | 6.339 |  |  | this paper |
| **RP73** | CF-patient | 6.342 | 66.5 | 5,975 |  |
| **PAO1** | Wound | 6.264 | 66.6 | 5,570 |  |
| **PA14** | Clinical | 6.538 | 66.3 | 5,892 |  |
| **LESB58** | CF-patient | 6.602 | 66.3 | 5,925 |  |
| **PA7** | Clinical | 6.588 | 66.5 | 6,286 |  |
| **M18** | Rhizosphere | 6.327 | 66.5 | 5,684 |  |

* draft genomes
