# Supplementary material for: Comparative genomics and biological characterization of sequential Pseudomonas aeruginosa isolates from persistent airways infection
Source: BMC Genomics. 2015 Dec 29;16:1105. doi: 10.1186/s12864-015-2276-8 (PMC4696338; doi:10.1186/s12864-015-2276-8)
Supplement: Additional file 4: — MLST typing of RP isolates [ 55 ]. (DOC 28 kb) [file 12864_2015_2276_MOESM4_ESM.doc]

**Additional File 4: MLST typing of RP isolates.**

**Table 2: MLST typing of RP isolates.**

| **Strain** | **Profile** | **acs** | **aro** | **gua** | **mut** | **nuo** | **pps** | **trp** |
| --- | --- | --- | --- | --- | --- | --- | --- | --- |
| RP1 | 395 | 6 | 5 | 1 | 1 | 1 | 12 | 1 |
| RP45 | 198 | 11 | 5 | 11 | 11 | 3 | 27 | 7 |
| RP73 | 198 | 11 | 5 | 11 | 11 | 3 | 27 | 7 |

*In silico* MLST typing was performed using Pubmlst (http://pubmlst.org) and Srst2 .
